# Initial Psychiatric Manifestations in Lewy Body Dementia: A Case Report

**DOI:** 10.1192/j.eurpsy.2025.1698

**Published:** 2025-08-26

**Authors:** M. Calvo Valcárcel, M. A. Adreo Vidal, C. Rodriguez Valbuena, M. Fernández Lozano, Ó. Martín Santiago, O. M. Segurado Martín, M. B. Arribas Simón, J. L. Cáceres Pereira, J. C. Fiorini Talavera, M. P. Pando Fernández, P. Martínez Gimeno, N. Navarro Barriga, M. J. Mateos Sexmero, B. Rodriguez Rodriguez, G. Lorenzo Chapatte, M. Ríos Vaquero, L. Rojas Vazquez, A. Monllor Lazarraga, L. Del Canto Martínez, F. J. Gonzalez Zapatero, M. D. L. Á. Guillén Soto, A. Aparicio Parras, L. Sobrino Conde, M. Queipo de Llano de la Viuda

**Affiliations:** 1 Hospital Clinico Universitario de Valladolid, Valladolid, Spain

## Abstract

**Introduction:**

Lewy Body Dementia (LBD) is the second most common neurodegenerative disease, after Alzheimer’s disease. Initial neuropsychiatric manifestations such as depression, delusions and hallucinations are frequently observed and sometimes make it difficult to diagnose the neurocognitive disorder underlying the symptoms, so it is important to perform a proper clinical examination, as the use of certain neuroleptics may worsen neurological symptoms.

**Objectives:**

This case aims to investigate the psychiatric clinical features of Lewy body dementia from a clinical and therapeutic perspective.

**Methods:**

A comprehensive search on psychiatric manifestations that may cover up dementia.

**Results:**

71-year-old female with depressive symptoms for the last 8 years. She is admitted to a psychiatric inpatient unit due to worsening of depressive symptoms despite correct adherence to treatment. Her psychiatric history includes a diagnosis of specific phobia, obsessive-compulsive disorder and depressive episodes with inhibitory symptomatology.

During her stay at the hospital, the patient is inhibited, perplexed and experiences feelings of embarrassment and guilt, along with persistent insomnia and poor response to different lines of treatment. Initially, there is notable intolerance to antipsychotics, resulting in worsening of motor and cognitive functions, as well as hypotension, using risperidone and olanzapine. After the withdrawal of treatment, the patient begins to exhibit delusional ideas and visual hallucinations, leading us to consider that she may be suffering from depression associated with an undiagnosed organic brain pathology.

Clinical tests (MoCA,MMSE) reveals cognitive symptoms which, along with the motor symptoms, suggests a Parkinson’s-dementia complex.

A PET-CT scan with fluorodeoxyglucose-F18 reveals severe hypometabolism in the left parietotemporal and prefrontal regions. These findings are consistent with LBD.

Treatment is initiated with rivastigmine and quetiapine. However, due to the presence of hypotension, quetiapine is replaced with clozapine 25 mg, resulting in a slight improvement in rest and affective responses to the psychotic symptoms.

**Conclusions:**

This case illustrates how depression and psychotic symptoms can serve as early indicators of dementia, stemming from the loss of dopaminergic and acetylcholinergic pathways as part of the neurodegenerative process.

These patients may present with a range of cognitive, neuropsychiatric, sleep, motor, and autonomic symptoms. Depression is prevalent in approximately 28% of these patients. Currently, clinicians diagnose LBD based on the presence of core clinical features and indicative biomarkers. Treatment can be complicated by patients’ sensitivity to certain medications, needing careful evaluation of potential side effects. Current guidelines recommend the use of antipsychotics such as quetiapine or clozapine at low doses, as these have a reduced risk of extrapyramidal effects.

**Disclosure of Interest:**

None Declared

